# Pediatric wrist fractures: variations in management across countries. An evidence-based summary of evidence

**DOI:** 10.1093/bmb/ldae014

**Published:** 2024-10-04

**Authors:** Luca Labianca, Cosma Calderaro, Nicola Maffulli

**Affiliations:** Department of Orthopaedic and Traumatology, S. Andrea Hospital, Faculty of Medicine and Psychology “Sapienza” University of Rome, Via di Grottarossa, 1035 00189 Rome, Italy; Department of Orthopaedic and Traumatology, S. Andrea Hospital, Faculty of Medicine and Psychology “Sapienza” University of Rome, Via di Grottarossa, 1035 00189 Rome, Italy; Department of Orthopaedic and Traumatology, S. Andrea Hospital, Faculty of Medicine and Psychology “Sapienza” University of Rome, Via di Grottarossa, 1035 00189 Rome, Italy; Centre for Sports and Exercise Medicine, Barts and the London School of Medicine and Dentistry, Mile End Hospital, Queen Mary University of London, Bancroft Rd, London E1 4DG, United Kingdom; School of Pharmacy and Bioengineering, Keele University School of Medicine, Staffordshire, Stoke-on-Trent ST4 7QB, United Kingdom

**Keywords:** pediatrics, wrist fractures, management, variability

## Abstract

**Introduction:**

Fractures of the distal radius are common in pediatric population, with considerable variation in the management of pediatric wrist fractures across different countries. It is crucial to consider the different approaches to conservative management and surgical intervention. The decision on the appropriate treatment method often depends on the type and severity of the fracture, as well as the available healthcare resources and expertise in each country. This article tries to identify these variations, so the various healthcare systems can work toward implementing best practices in the management of pediatric wrist fractures on a global scale.

**Source of data:**

Published peer-reviewed articles identified in electronic databases, including PubMed Scopus and Google Scholar.

**Areas of agreement:**

The management of pediatric wrist fractures can differ significantly among countries given the high variability in healthcare resources and cultural practices.

**Areas of controversy:**

The management of pediatric wrist fractures can be challenging in certain countries, especially in developing regions with limited resources.

**Growing points:**

Challenges such as long therapeutic delays, lack of appropriate anesthesia, and the absence of fluoroscopy can complicate the treatment process. Randomized controlled clinical trials (RCTs) are vital in providing high-quality evidence to guide clinical decision-making, especially in the field of pediatric wrist fractures.

**Areas timely for developing research:**

Efforts to support and prioritize the conduct and dissemination of RCTs in pediatric wrist fracture management can ultimately lead to more consistent, effective, and evidence-based care for children with wrist fractures worldwide.

## Introduction

Fractures of the distal radius are very common [1], with ~70 000 fractures of the distal forearm per year in the UK [2].

Pediatric wrist fractures, particularly those involving the distal radius, are the most common fractures in children [3,4]. Approximately 3–9% of all athletic injuries occur in the hand or wrist, and are more common in adolescent athletes than adults [5,6]. These fractures can vary in severity, with buckle fractures [7,8] being stable and usually not displaced, while greenstick fractures and other displaced fractures may require some intervention [9–11]. Malrotation of the distal radius is common following distal radius fracture [12].

There is considerable variation in the management of pediatric wrist fractures across different countries [13–22]. Particularly in Western countries, super-specialization has resulted in an increased number of surgeries performed as well as health care costs [23].

The overall complication rate following internal plate fixation of distal radius fractures in the literature varies from 4 to 36%. 3D-printed models for preoperative planning surgically treating DRF AO/OTA C type can help minimize the complication rate [24].

Understanding the variations in pediatric wrist fracture management across different countries is essential to gain insights into global disparities and identify areas for potential improvement in healthcare delivery for pediatric orthopedic injuries. In this comparative analysis, we delve into the specific management practices and outcomes of pediatric wrist fractures in different countries, shedding light on the existing challenges and opportunities to enhance pediatric orthopedic care on a global scale.

When comparing the management of pediatric wrist fractures across various countries, it is crucial to consider the different approaches to conservative management and surgical intervention [25]. The decision on the appropriate treatment method often depends on the type and severity of the fracture, as well as the available healthcare resources and expertise in each country [26].

Conservative management of pediatric wrist fractures typically involves the use of casts or splints to immobilize the affected wrist and promote healing. This approach is commonly preferred for stable fractures such as buckle fractures, where the bones are not displaced. However, there may be variations in the duration of immobilization and the type of casting materials used across different countries [13–22].

On the other hand, surgery may be necessary for more severe or displaced fractures, especially those that involve the distal radius. Surgical techniques such as closed reduction and percutaneous pinning may be employed to realign the fracture and provide stability during healing [27]. The choice of surgical intervention and the specific techniques used can differ according to the orthopedic expertise and available medical technology in each country.

Understanding the diverse treatment approaches and outcomes for pediatric wrist fractures across different countries can offer valuable insights into the global disparities in pediatric orthopedic care. By identifying these variations, healthcare systems can work toward implementing best practices and addressing the specific challenges associated with the management of pediatric wrist fractures on a global scale.

## Materials and methods

**Figure 1:**
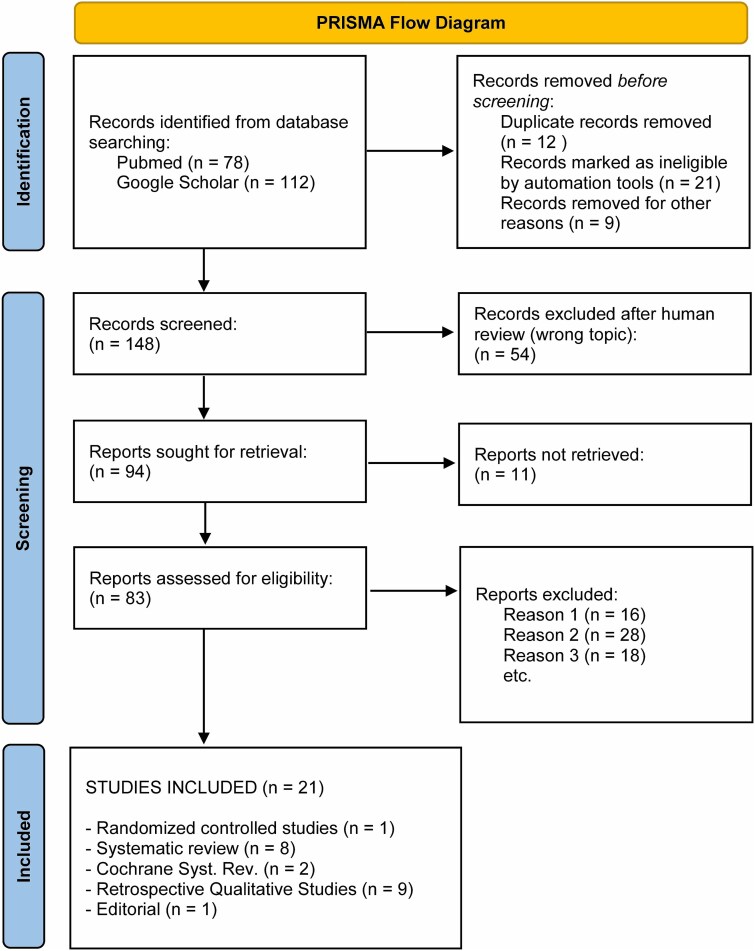
Prisma flow diagram.

To conduct the comparative analysis of pediatric wrist fracture management across different countries, a literature review was conducted using published peer-reviewed articles (Fig. 1—Prisma diagram). The search terms used were “pediatric wrist fractures” OR “children wrist fractures” OR “juvenile wrist fractures”) AND (management OR treatment OR therapy) AND (comparison OR comparative analysis) AND (“healthcare system” OR “country comparison” OR “international study”). The literature search was conducted using electronic databases, including PubMed Scopus and Google Scholar. Only English-language articles were included in the review.

## Results

On PubMed, were found a total of 78 articles related to the search terms; Google Scholar, listed 112 relevant articles.

After reviewing all the text of the articles, 21 of them were included in the present work. These articles cover a wide range of topics, including cultural influences on pediatric fracture management, treatment strategies for pediatric distal radius fractures, comparative studies on global differences in pediatric wrist fracture management, and the integration of traditional and modern healthcare practices in the management of pediatric wrist fractures.

The articles retrieved included systematic reviews, comparative studies, observational research, and qualitative analyses. The level of evidence in the selected articles ranges from Level I to Level IV, based on the Oxford Centre for Evidence-Based Medicine Levels of Evidence. The systematic reviews and meta-analyses identified in the search provide a high level of evidence, as they critically appraise and synthesize existing research findings on pediatric wrist fracture management worldwide. These articles offer comprehensive evaluations of treatment strategies and outcomes, contributing valuable insights to evidence-based practice.

Additionally, comparative studies on global differences in pediatric wrist fracture management and cultural influences on treatment approaches provide evidence at Level II and III, respectively. These studies compare various treatment modalities and healthcare practices across different regions and cultures, shedding light on the impact of sociocultural factors on healthcare decision-making.

Furthermore, observational research and qualitative analyses regarding the integration of traditional and modern healthcare practices in pediatric wrist fracture management contribute Level III and IV of evidence, pointing to the complexities of incorporating traditional healers’ treatments into the overall healthcare system and the implications for patient care.

## Discussion

There is a definite seasonal variation in pediatric orthopedic trauma, or, better, a relationship with time and climate [28]. One might expect that in countries with warmer climates, as more outdoor activities can be carried out, there would be a higher incidence of pediatric trauma.

In the USA, the treatment approach for pediatric wrist fractures typically involves a combination of manual reduction and casting. After manual reduction, a cast is applied to immobilize the fracture and promote healing [11]. A Canadian study focused on buckle fractures and concluded that splinting in the Emergency Department with primary care follow-up appears to be a reasonable management strategy for these fractures [16].

In the UK, the management of pediatric wrist fractures may also involve manual reduction and casting, but there is a growing trend toward using removable splints as an alternative to traditional casting. The use of removable splints allows early mobilization and may reduce the inconvenience associated with cast immobilization [7,8,10,11]. In Sweden, the treatment of pediatric wrist fractures follows a similar approach to that in the UK [11]. Manual reduction and casting are commonly used, but there is also a focus on early mobilization with removable splints. In Switzerland, the management of pediatric wrist fractures also involves manual reduction and casting [11]. However, there is a greater emphasis on surgical interventions and internal fixation, such as intramedullary nails or plating, particularly for more complex fractures [27].

In Asia, the management of pediatric wrist fractures varies significantly across different countries, reflecting the diverse healthcare systems and resources available in the region [8]. Focusing on the South-East Asian countries and excluding other regions such as the Middle East and Russia with a very different cultural and economic background several issues need to be highlighted [20].

In Japan, a leading advanced economy, the management of pediatric wrist fractures often involves a combination of conservative management and surgical intervention. Similar to the approaches in the USA and some European countries, manual reduction and casting are frequently employed [18]. However, there is also a growing trend toward applying advanced surgical techniques for more severe fractures, aligning with the country’s advanced healthcare expertise and technology.

The emerging economies in South-East Asia, particularly in the newly industrialized countries, such as Malaysia, Thailand, the Philippines, India, and China, offer limited data regarding the specific approaches to pediatric wrist fracture management [18]. The most recent data available suggest the need for further studies and understanding of the treatment practices and outcomes in these rapidly developing economies.

In the USA, the surgeon–patient interaction regarding the treatment of pediatric wrist fractures typically involves a collaborative approach, with patients being more independent and actively involved in the decision-making process. Surgeons present various treatment options, including manual reduction and casting, and provide detailed explanations to guide patients in making informed decisions about their child’s treatment. Ultimately, the decision regarding the choice of treatment is driven by the patient’s preferences and surgeons work to accommodate those preferences within the bounds of medical necessity [4,11].

On the other hand, in many Asian countries, including those in South-East Asia, the dynamics of the surgeon–patient interaction for pediatric wrist fracture management differ significantly. Patients in these countries often prefer a more guided paternalistic approach, where the surgeon’s recommendations carry significant weight in the decision-making process. The discussion around treatment options is typically limited, and the surgeon’s expertise plays a pivotal role in determining the course of action. This cultural inclination toward implicit obedience to the surgeon’s guidance is a key factor shaping the interaction between the medical provider and the patient [18].

With the ongoing modernization and advancement of healthcare systems in Asian countries, particularly in emerging economies, there is a gradual shift in the dynamics of surgeon–patient interactions. Younger patients and their families are beginning to question and seek more active involvement in the decision-making process, aligning more closely with the approach seen in Western countries. As a result, there is an evolving trend toward greater patient autonomy and participation in treatment decisions, reflecting the influence of changing societal norms and increasing access to healthcare information.

To further understand the nuances of surgeon–patient interactions and their impact on pediatric wrist fracture management in Asian countries, continued research and observation of these evolving trends will be essential. This ongoing examination will provide valuable insights into the cultural and healthcare dynamics that shape the decision-making process in pediatric orthopedic care.

Traditional healers play a substantial role in the healthcare system of sub-Saharan Africa, particularly in the management of fractures. In many sub-Saharan countries, fractures are often managed by traditional bone-setters, who offer their services to the entire population [14]. The World Health Organization has recognized the importance of traditional bone-setting, especially considering the increasing shortage of medical services from the brain drain in the region. This scarcity of medical services makes it advisable for healthcare policymakers to consider integrating traditional bone-setting into the overall healthcare system.

Despite the recurrent argument that traditional bone-setters’ treatments are not formally organized, the available data reveal that the methods, materials, and follow-up offered by different bone-setters are quite similar. This suggests that their treatments are comparable to methods described in the past and are widely available to this population. However, there is a lack of systematic reviews on the methods and results of bonesetter treatment, highlighting the need for further research in this area.

A study conducted in sub-Saharan Africa, Yaoundé, Cameroon, found that non-operative management was the most common approach for pediatric wrist fractures (130 of 147 fractures), with some requiring manipulations under anesthesia or open reductions with internal fixation (29.3 and 17%, respectively) [19].

As the healthcare landscape in sub-Saharan countries continues to evolve, the cooperation between modern medical practices and traditional bone-setting should be further explored. Understanding the effectiveness and outcomes of traditional bone-setting for fracture management can provide valuable insights for healthcare policymakers and practitioners. By recognizing the role of traditional healers and their contributions to healthcare in sub-Saharan Africa, strides can be made toward providing inclusive and effective healthcare for all members of the population.

In the United Arab Emirates (UAE), the treatment of pediatric wrist fractures reflects a strong connection between culture, religion, and healthcare practices. Many mothers in the UAE opt for cultural remedies as their first line of defense against illnesses given the practices’ strong foundations in their cultural heritage. Herbal remedies, Quranic healing, and other traditional methods are perceived to be both effective and spiritually comforting, reinforcing participants’ sense of cultural identity [13].

However, there are unintended consequences of relying on traditional treatments, as reported in other regions. The use of ineffective remedies may result in delays in seeking appropriate medical care for children, potentially compromising their health [13].

Furthermore, misconceptions regarding the safety and efficacy of traditional remedies have been identified in the UAE, emphasizing the need for evidence-based healthcare education. Considering this, there is an opportunity to integrate traditional cultural practices with evidence-based healthcare to ensure the well-being of children with wrist fractures in the UAE. It is essential to provide education and awareness programs that emphasize the importance of seeking appropriate medical care while respecting cultural practices. Balancing traditional beliefs with proven medical interventions can lead to improved outcomes and better healthcare decision-making for pediatric wrist fractures in the UAE.

The management of pediatric wrist fractures often involves manual reduction followed by casting. However, there is also a trend toward using alternative methods, such as percutaneous pinning or external fixation, especially in more complex fractures. In Italy, the treatment of pediatric wrist fractures generally involves manual reduction and casting. However, there is also a growing interest in utilizing removable splints as an alternative to traditional casting, allowing for early mobility and potentially greater convenience. Overall, there is wide variation in the management of pediatric wrist fractures across different countries. These variations may be influenced by factors such as resources, healthcare infrastructure, cultural preferences, and advancements in surgical techniques [11,22,23,25,27].

## Conclusions

The management of pediatric wrist fractures can differ significantly among countries given the high variability in healthcare resources and cultural practices. Factors such as access to specialized healthcare facilities, availability of trained orthopedic surgeons, cultural beliefs, and socioeconomic factors can influence the choice between conservative management and surgical intervention. For example, in sub-Saharan Africa, where resources for specialized pediatric fracture care may be limited, non-operative management is often the mainstay of treatment [19].

The management of pediatric wrist fractures can be challenging in certain countries, especially in developing regions with limited resources. Challenges such as long therapeutic delays, lack of diligent anesthesia, and the absence of fluoroscopy can complicate the treatment process. These challenges can result in suboptimal outcomes and potentially higher rates of complications. Additionally, cultural beliefs and practices can also pose challenges in the management of pediatric wrist fractures.

The dynamics of surgeon–patient interactions in Asian countries, the role of traditional bone-setters in sub-Saharan Africa, and the influence of cultural remedies on healthcare practices in the UAE all contribute to the diversity of approaches in pediatric wrist fracture management. Furthermore, understanding the effectiveness and outcomes of traditional bone-setting for fracture management in sub-Saharan Africa, as well as balancing traditional beliefs with proven medical interventions in the UAE, can guide healthcare policymakers and practitioners toward providing inclusive and effective healthcare for all members of the population. By acknowledging the influence of cultural preferences and advancements in surgical techniques, healthcare providers can tailor treatment approaches to best serve the needs of pediatric patients with wrist fractures.

Randomized controlled clinical trials (RCTs) are vital in providing high-quality evidence to guide clinical decision-making, especially in the field of pediatric wrist fractures. The findings of RCTs can significantly influence treatment choices and clinical practice by providing definitive evidence on the efficacy and safety of different management approaches. For instance, RCTs comparing non-operative management with surgical interventions for pediatric wrist fractures can offer insights into the optimal approach in different clinical scenarios, leading to more standardized and evidence-based practices [8,10,11,18,23,25,27].

The implementation of findings from RCTs has the potential to impact clinical practice by improving patient outcomes, reducing complications, and standardizing treatment protocols. Moreover, it can aid in addressing the variations in pediatric fracture management observed across different countries. By disseminating and incorporating the findings of RCTs into clinical practice, healthcare providers can offer more consistent and evidence-based care to pediatric patients with wrist fractures, irrespective of the healthcare resources and cultural beliefs prevalent in their region.

Additionally, the insights gained from RCTs can contribute to the development of guidelines and protocols tailored to the specific needs and challenges faced in diverse clinical settings. This adaptable approach to pediatric fracture management, informed by robust evidence from RCTs, has the potential to bridge the gaps in care and elevate the standard of treatment globally.

A few words need to be spent about prevention. As most pediatric wrist fractures occur in the home environment, efforts should be directed to reduce childhood injuries in the home. In developing countries, most parents go to work and leave their younger children (infants and toddlers) under the care of their elder siblings. Provision of crèches and supervised nurseries at workplaces could reduce such accidents [29]. The success of the notable “Kids can’t fly” campaign developed by the New York health department highlights the effectiveness of simple accident prevention programs in the peri domestic environment [30].

In conclusion, the impact of different RCTs in the field of pediatric wrist fracture management cannot be overstated. Their contribution to shaping clinical practice, improving patient outcomes, and standardizing treatment approaches is crucial to address the variations observed across countries. Efforts to support and prioritize the conduct and dissemination of RCTs in pediatric fracture management can ultimately lead to more consistent, effective, and evidence-based care for children with wrist fractures worldwide.

As the healthcare landscape continues to evolve, it is essential to recognize the importance of integrating traditional practices with evidence-based medical care. This integration can lead to improved outcomes and better decision-making, ensuring the well-being of children with wrist fractures. Additionally, ongoing research and observation of evolving trends in surgeon–patient interactions will provide valuable insights into the cultural and healthcare dynamics that shape the decision-making process in pediatric orthopedic care.

## Data Availability

Not applicable.
